# A rare case of pseudotumor cerebri in adult Lyme disease

**DOI:** 10.1002/ccr3.2582

**Published:** 2019-12-18

**Authors:** Louise Nørreslet Gimsing, Line Sofie Lunde Larsen

**Affiliations:** ^1^ Department of Specialized Neurorehabilitation Hvidovre Hospital Hvidovre Denmark; ^2^ Neurological Department Herlev Hospital Herlev Denmark

**Keywords:** chronic borreliosis, chronic infection, Lyme disease, pseudotumor cerebri

## Abstract

It is important to recognize the rare manifestations of chronic Lyme disease to prevent permanent disabilities. We present an adult case of chronic Lyme disease, who developed pseudotumor cerebri and who needed supplementary surgical treatment. We compare it to the existing published literature, reviewed by a systematic approach.

## Graphical abstract

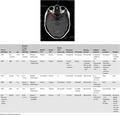

## 1 INTRODUCTION

The chronic phases of infection with the spirochete Borrelia burgdorferi (Bb), European Lyme disease, are characterized by the involvement of several organ systems. In the nervous system, neuroborreliosis can develop in untreated individuals within 2‐6 weeks^1^ and includes signs of meningeal irritation with nuchal tenderness, fatigue, nausea, and the two cardinal symptoms: painful meningoradiculitis and peripheral motor deficits (Bannwarths triad).^1^

Chronic neuroborreliosis (duration >6 months) can have numerous presentations.

We here present a rare case of chronic neuroborreliosis seemingly presenting as idiopathic intracranial hypertension (IIH) or pseudotumor cerebri (PTC) in a previously healthy woman. A case needed both antibiotic and neurosurgical treatments.

## 2 PRESENTATION

A 51‐year‐old woman with no previous medical history was admitted to our neurological clinic on suspicion of IIH. For about 1 year, she had experienced slowly progressive fluctuating headache, bilaterally located, throbbing, from low to moderate in intensity. Within the last 3 months, the headache had increased in intensity. Moreover, she described nausea, occasional vomiting, light dizziness, discrete tinnitus, and unintended weight loss of 10 kg. Five months prior to admission, she had started noticing a blurred disturbance of the visual field in her left eye. Due to the tinnitus and dizziness, an ear, nose, and throat (ENT) doctor booked her a magnetic resonance imaging (MRI) of cerebrum. This showed a partial empty sella, meningeal enhancement, and distended optical nerve sheaths, suggestive of increased intracranial pressure. Shortly after, she was evaluated at the ophthalmological clinic. Here, she was diagnosed with bilateral chronic papilledema, bilateral visual field impairment and on the left eye reduced color vision, and a visual acuity of 3/6. This caused a direct admission to our neurological department, where she could tell of a debut 1.5 years before of moderate neck pain and upper backpain, but no recollection of insect bite or rash.

## 3 ASSESSMENT

On neurological examination, she had slight problems of walking in a straight line, but otherwise performed normally aside from the vision loss.

## 4 DIAGNOSIS AND MANAGEMENT

**Figure 1:**
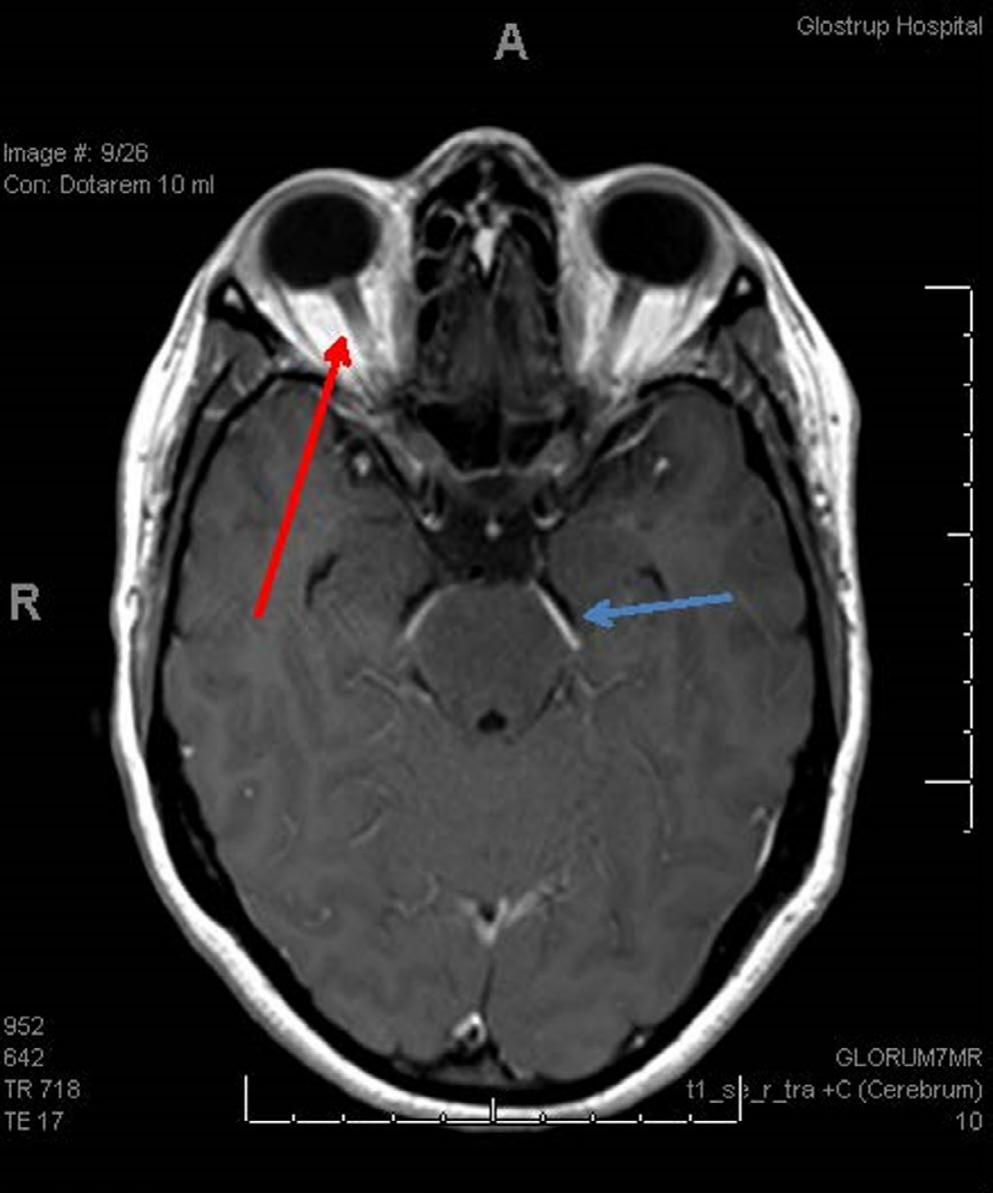
MRI of cerebrum with gadolinium contrast, axial picture. Red arrow showing papilledema and blue arrow showing meningeal enhancement

A laboratory investigation, for example, complete blood cell count, C‐reactive protein (CRP), electrolytes, liver enzymes, albumin, creatinine, lactate dehydrogenase, and thyroid‐stimulating hormone, was within normal limits. A computed tomography (CT) scan of the cerebrum excluded sinus thrombosis, while a repeated MRI showed postcontrast leptomeningeal enhancement and a normal‐sized ventricular system (Figure 1). A lumbar puncture (LP) was subsequently performed with an opening pressure of 500 mm H_2_O.

The cerebrospinal fluid (CSF) showed an increased protein count (306 mg/dL [20‐40 mg/dL]), positive oligoclonal bands, an increased lymphocytic pleocytosis (77 U/mm,^2^ 93% lymphocytes), unspecified IgG >300 mg/L, and Bb‐specific IgG >2.36 mg/L, while Bb‐specific IgM was negative. CSF analysis for viruses was negative, and supplementary blood analysis for HIV, tuberculosis (quantiferon test), ACE, ANA, and ANCA was all normal.

The patient was initially treated with intravenous (iv) Ceftriaxone daily and Acetazolamide.

After one week of treatment, the symptoms worsened, and therapeutic repeated LP was made with good symptom relief. The effect was though temporary, and consequently, about 3 weeks after admission, a ventricular peritoneal shunt (VPs) had to be implanted, which stopped the progression of the symptoms.

Six months after the ended 18 days of antibiotic treatment (AB), the head pain and neck pain, as well as nausea and vomiting, were gone. Subjectively, the visual acuity and visual field defects had improved, but objectively, a central scotoma, lack of color vision, and atrophy of the optic nerve were still present.

## 5 COMMENT

We describe a rare presentation of adult neuroborreliosis.

Nord and Karter^3^ describe in 2003 “the first case” of PTC as a complication to Lyme disease in adults, but already in a review^2^ from 1986, Burgdorfer et al describe a case with positive Bb antibody titer with papilledema and increased opening pressure at LP.

Using the database PubMed, a search of the combinations of “borrelia,” “borreliosis,” “Lyme,” “intracranial hypertension,” and “pseudotumor cerebri” revealed only 5 previously published cases in adults (Table 1).^2^, ^3^, ^4^, ^5^, ^6^ In the same database search, we found 35 cases in children between 4 and 14 years old, the first described in 1985.

**Table 1:** The table shows the main characteristics of the case of this article and the 5 previous published cases of pseudotumor cerebri in Bb‐infected adults in chronological order

Year of publication	Sex	Age	Duration of symptoms at admission	Papilledema	Bb IgM CSF	Bb IgG CSF	Bb IgG CSF/serum‐ratio	Pleocytosis (leukocytes)	CSF‐protein	Opening pressure	Antibiotic treatment	Other treatment	Sequelae
1986^1^	Not reported	>32 y	Not reported	Bilateral (fundoscopy)	Positive	Positive	Elevated.	Not reported	Not reported	420 mm H_2_O	Iv Penicillin, 2 MIE for 10 d	Not reported	atrophy of optic nerve.
2003^3^	Male	37 y	5 wk	Not present (fundoscopy)	Positive	Positive	Elevated.	1 U/mm^3^ (after 3 wk of treatment)	Normal	Could not be measured. CSF was shot through the room	P.o. Doxycycline 100 mg. ×2 daily in 3 wk	2 wk after three therapeutic lumbal punctures with 4 and 6 d in between	None.
2003^2^	Female	19 y	8 wk (2 mo)	Bilateral (fundoscopy)	Not reported	Positive	1.1	Not reported	Not reported	300 mm H_2_O	Iv 2 g Ceftriaxone for 2 wk	None.	None.
2008^4^	Male	34 y	Almost a week (6 d)	Bilateral (fundoscopy)	Positive	Negative	Not reported	6 U/mm^3^	100 mg/dL	260 mm H_2_O	Iv 2 g. Ceftriaxone for 4 weeks	None	None
2016^5^	Male	20 y	1 wk	Bilateral (fundoscopy)	Positive	Negative	Not reported	14 U/mm^3^	37 mg/dL (normal)	400 mm H_2_O	Iv 2 g. Ceftriaxone for 4 wk	Acetazolamide	None.
2017 (The case described in this article)	Female	51 y	78 wk (1.5 y)	Bilateral (fundoscopy and MRI)	Negative	Positive	2.36	77 U/mm^3^	300 mg./dL	500 mm H_2_O	Iv 2 g. Ceftriaxone for 18 d	Acetazolamide 1 wk, 4 therapeutic LBP, VP‐shunt	On one eye papilla optica atrophy, loss of color vision, and a central scotoma.

Abbreviation: Bb, Borrelia burgdorferi.

ΡTC in children in relation to Lyme disease has been described as a rare entity (1a, 1b), yet the literature shows that the entity is even more rare (or underdiagnosed) in adults. Literature has suggested a different presentation of Lyme disease in children compared to adults in, for example, concern to the involvement of neurological symptoms,^1^, ^7^ but no study has proved the mechanism behind this. Thus, it remains a hypothesis.

Nonetheless, this article has chosen to go into detail with the adult cases published.

The presentation of pseudotumor cerebri is usually headache, transient or gradual visual loss, photophobia, diplopia (sometimes with 6th cranial nerve palsy), weight loss, nausea or vomiting, and with papilledema (unilateral or bilateral) often, but not always present.

The term used for the condition is previously discussed^3^, ^6^: the reason being that the definition of PTC initially required a normal CSF. The presence of pleocytosis would therefore better suggest the term secondary intracranial hypertension. Though in all the 5 published cases, the term PTC is used and so will we in this article.

The mechanism remains unknown, which is suggested to be an inflammatory response to the chronic Bb meningitis, impairing the CSF reabsorption in the arachnoid villi.^3^, ^8^

In pediatric literature, corticosteroids,^9^ therapeutic LP, VPs,^9^ and acetazolamide^8^, ^10^ have been suggested as supplementary treatments to AB of the Bb‐induced PTC. Later studies have confirmed that treatment with corticosteroids does not belong in the treatment of any form of Lyme's disease.^11^

The rationale behind the therapeutic LP and the VPs comes from the evidence and use of these methods in medication‐resistant IIH patients.^12^ Therefore, both were performed when AB did not work fast enough to avoid further symptom progression.

The many pediatric cases of Bb‐induced PTC point to an overall good prognosis^10^ once AB has been initiated, and the previously published adult cases (Table 1) show a similar pattern. The case presented here is the first adult case with a need for surgical intervention to decrease the intracranial pressure on top of the AB.

The combination of negative Bb IgM and positive IgG in CSF also points to a chronic infection in contrary to the other published cases.^1^ The positive oligoclonal bands in CSF are an unspecific inflammatory biomarker for intrathecal synthesis of IgG, as thus by default does not differ between an infectious and a non‐infectious etiology. Specific inflammatory and infectious diseases have been found to be more correlated to positive oligoclonal bands. In the case of neuroborreliosis, previous studies have shown that up to 74% had positive oligoclonal bands at a time of diagnosis.^13^

The positive result of the test in our case, itself, did not give reason to rule out supplementary infectious or inflammatory diseases, for example, test for multiple sclerosis.

The patient ended up having permanent visual impairment despite treatment, which illustrates the importance of remembering this rare differential diagnosis of secondary intercranial hypertension/PTC to be able to initiate treatment as early as possible.

## CONFLICT OF INTEREST

None declared.

## AUTHOR CONTRIBUTIONS

Louise Nørreslet Gimsing, MD: contributed to the design of the case study, the literature revision, and the writing of the manuscript. Line Sofie Lunde Larsen MD: contributed with the choice of case, and to the critical feedback and supervision in the writing of the manuscript.

## PATIENT CONSENT

The patient of whom the case report concerns has been informed about the publication and declared the consent orally and by a written formula.

## Data Availability

Raw data were generated at the patient registry of the public hospitals in Denmark. Derived data supporting the findings of this study are available from the corresponding author LNG on request.
